# Inference of immune cell composition on the expression profiles of mouse tissue

**DOI:** 10.1038/srep40508

**Published:** 2017-01-13

**Authors:** Ziyi Chen, Anfei Huang, Jiya Sun, Taijiao Jiang, F. Xiao-Feng Qin, Aiping Wu

**Affiliations:** 1 Center for Systems Medicine, Institute of Basic Medical Sciences, Chinese Academy of Medical Sciences & Peking Union Medical College, Beijing 100005, Suzhou Institute of Systems Medicine, Suzhou, Jiangsu 215123, China

## Abstract

Mice are some of the widely used experimental animal models for studying human diseases. Defining the compositions of immune cell populations in various tissues from experimental mouse models is critical to understanding the involvement of immune responses in various physiological and patho-physiological conditions. However, non-lymphoid tissues are normally composed of vast and diverse cellular components, which make it difficult to quantify the relative proportions of immune cell types. Here we report the development of a computational algorithm, ImmuCC, to infer the relative compositions of 25 immune cell types in mouse tissues using microarray-based mRNA expression data. The ImmuCC algorithm showed good performance and robustness in many simulated datasets. Remarkable concordances were observed when ImmuCC was used on three public datasets, one including enriched immune cells, one with normal single positive T cells, and one with leukemia cell samples. To validate the performance of ImmuCC objectively, thorough cross-comparison of ImmuCC predicted compositions and flow cytometry results was done with in-house generated datasets collected from four distinct mouse lymphoid tissues and three different types of tumor tissues. The good correlation and biologically meaningful results demonstrate the broad utility of ImmuCC for assessing immune cell composition in diverse mouse tissues under various conditions.

Tissue-infiltrating immune cells play important roles in causing and resolving various disorders including cancer, infection and autoimmunity^1^,^2^,^3^,^4^. Mouse models have been widely used to investigate the function of different types of immune cells in tissues under different disease conditions owing to mice’s similarity to humans in physiology and anatomical structures^5^. For example, multiple lines of evidence from mouse models have suggested good correlations between the immune cell compositions of specific tissues and prognosis of various immune-related diseases^6^. Thus, characterizing tissue infiltration of immune cells would be highly useful towards quantifying immune responses inside the affected tissues and for better understanding the immunological mechanisms involved in disease development.

Based on their cell surface markers, immune cell types could be qualitatively and quantitatively measured via several experimental methods, including flow cytometry^7^, affinity purification^8^, and immunohistochemistry^9^. Using flow cytometry, Gunn *et al*. developed a protocol that could accurately quantify 11 immune cell types in non-lymphoid tissues^7^. McGuire *et al*.^9^ used immunohistochemistry to assess the differences in the number of intraepithelial CD8+ T cells between African Americans and Caucasians. Hurwitz *et al*. developed an antibody-based affinity purification method to purify tumor-resident leukocytes^8^. However, two major limitations hinder the routine use of these methods in the determination of immune cell compositions in large-scale samples. First, the fact that some types of immune cells lack suitable cell surface markers can cause them to be overlooked during detection^10^,^11^. Second, dissociating tissues into single cells for subsequent quantification of the proportions of them in complex tissues is highly demanding and prone to error.

To complement the histological and immunological approaches, several computational deconvolution methods have been developed to calculate the tissue immune cell fractions from human transcriptome data^12^,^13^. The principle underlying this computational strategy is that the gene expression profile of heterogeneous tissues is assumed to be a mixture of cell-type specific expression. In this way, the relative compositions of each type of immune cell in the tissue could be deconvolved from the mixed tissue expression profile^12^. Recently, a few computational methods have been developed for both human blood and tumor samples based on microarray and RNA-seq expression data^14^,^15^,^16^,^17^. Among these published methods, a nu-support vector regression (SVR) based method, CIBERSORT, showed significant advantages, specifically its tolerance to background noise introduced by other unknown cells^18^.

However, all these computational methods were developed for human blood or tumor samples. Despite the extreme importance of mouse models in the study of human disease, there is still no readily available computational method suitable for estimating the compositions of immune cells from mouse tissue expression profiles. Here, a mouse-specific algorithm called ImmuCC was developed to infer the proportions of 25 types of immune cells with 511 selected signature genes from mouse tissue microarray datasets. In total, the compositions of 25 types of mouse hematopoietic cells, including granulocytes, B cells, T cells, natural killer cells, dendritic cells and mono/macrophages, could be estimated using the ImmuCC algorithm. Good performance and robustness were observed not only with simulated datasets, but also with in-house generated validation datasets of enriched cell subsets, mouse immune organs and mouse tumor tissues, demonstrating the broad utility of ImmuCC.

## Results

### An overview of the ImmuCC model

**Figure 1:**
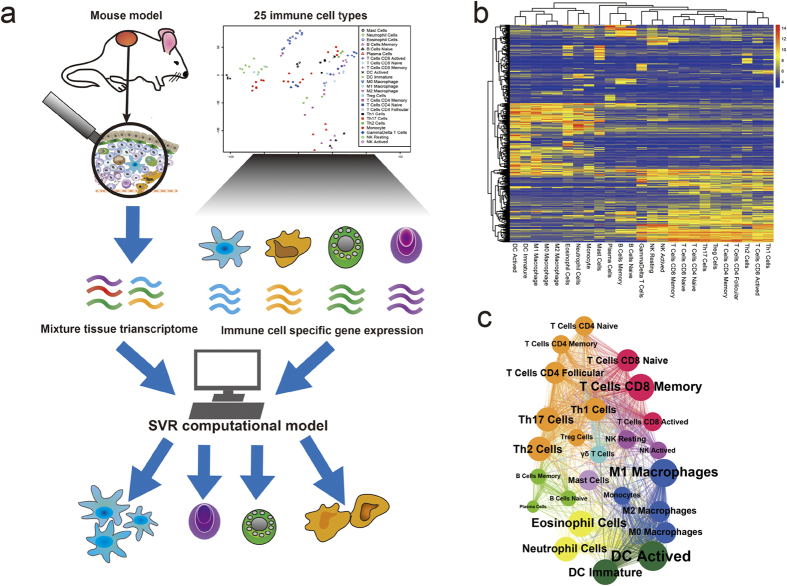
Schematics of estimating immune cell compositions from mouse tissue expression profile data. (**a**) An overview of the computational model ImmuCC. (**b**) Heatmap of signature matrix for clustering all 511 signature genes among 25 immune cell types. (**c**) Relationship of 25 immune cell types by shared signature genes. The cell types are colored differently, the dot areas are proportional to the number of signature genes.

The expression profile of a given tissue is considered as a hybrid ensemble of gene expressions across multiple cell subsets. In theory, the relative proportions of cell subsets, including 25 types of immune cells, can be deconvolved from the tissue expression profile (Fig. 1a). Here a computational model named ImmuCC was developed to infer the relatively compositions of 25 immune cell types with 511 signature genes via the linear support vector regression (SVR) approach. Three key steps were included in the ImmuCC model (Fig. 1a, see Methods for more details). First, 25 types of immune cells were identified via sample clustering and filtering (Supplementary Fig. S1). Second, 511 signature genes among all 25 immune cell types were selected to produce an input signature matrix for estimating the proportions of cell types (Fig. 1b). Third, the SVR approach was used to deconvolve the target mixture based on the input signature matrix. Then, to assess the robustness and availability of the system, the ImmuCC model was evaluated in three different datasets, including simulated datasets, enriched immune cell samples and tumor tissue samples.

### Construction of the signature matrix

The signature matrix consists of 511 genes that distinguish 25 mouse hematopoietic cell types, including six major cell types, granulocytes, B cells, T cells, natural killer cells, dendritic cells and mono/macrophages (Supplementary Fig. S2). To produce this signature matrix, raw expressed genes were filtered and run through four steps: (1) Raw data collection and preprocessing; (2) Calculation of Differential Gene Expression; (3) Filtering out non-hematopoietic genes; (4) Signature matrix iteration and gene selection (see Methods for more details).

In total, 115 datasets covering 25 immune cell types were kept after raw data preprocessing (Methods). Then, because some genes may have relatively high expression in both immune cells and non-immune cells, a non-hematopoietic gene filter was conducted. With the help of 348 arrays including 120 different tissues or cell lines for different tissue types, 5185 genes in Mouse genome 430 2.0 Array were treated as hematopoietic genes (Supplementary Fig. S3). Next, to prevent interference from genes highly expressed in tumors, probes with a mean log2 expression value >7 in non hematopoietic tumor tissues were here used as tumor specific expressed probes and removed from the signature matrix. Finally, 511 genes were selected to establish the signature matrix. Using these 511 signature genes for sample clustering, lymphocytes and myelocytes were clearly distinguished as expected (Fig. 1b). Some genes were also found to be shared by two even more cell types (Fig. 1b and c), which indicated not only cell type specific biomarkers, but also that genes shared among cell types play important roles in the signature matrix.

### Assessment of algorithm performance with the simulated datasets

**Figure 2:**
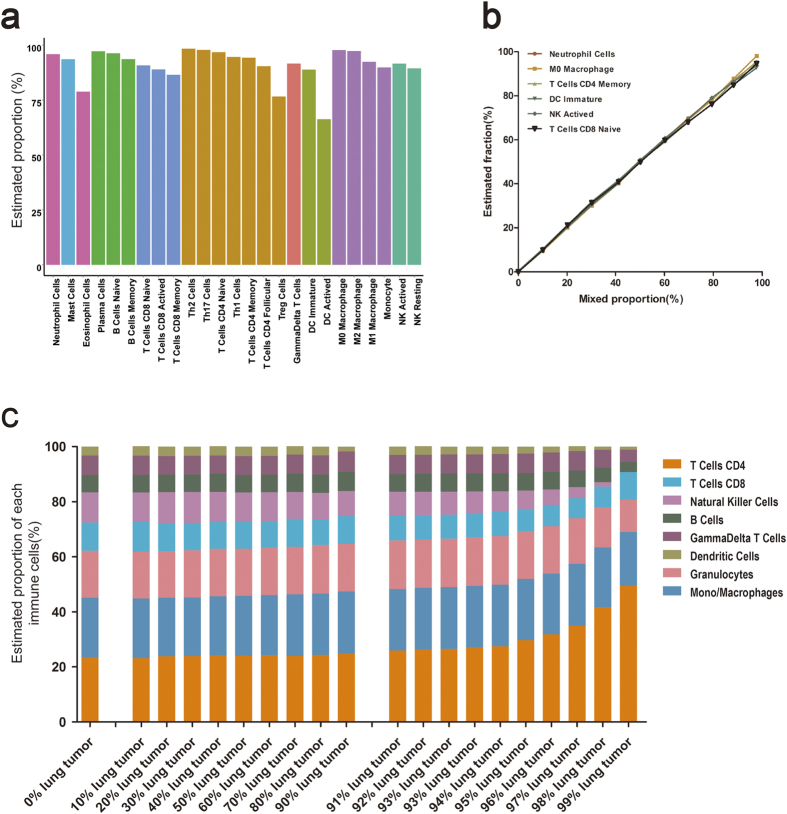
Model assessment in the simulated datasets. (**a**) Performance in the 25 individual cell types. Nine major cell types were colored differently. (**b**) Performance in the mixture of one target cell type with other multiple immune cells randomly. The proportion of the testing immune cell ranges from 0 to 100 percent. (**c**) Performance in the mixture of immune cells with tumor contents. The leftmost bar represents the real and reference immune cell compositions. The tumor mixture ranges from 0 to 99 percent.

To benchmark the sensitivity and accuracy of ImmuCC, in addition to the dataset with unique immune cell type, two more simulated datasets with well-defined compositions were generated, one including a mixture of multiple immune cell types and the other including a mixture of immune cells with tumor contents. The performance of ImmuCC was first evaluated against the training datasets containing single types of immune cells. As shown in Fig. 2a, the predicted proportions of most immune cell types were almost all greater than 90%, except the Treg and DC activated cells. Then a more common issue was addressed, whether multiple immune cell subtypes would affect the detection ability of the model. For each cell type, a background mixture consisting of the other 24 immune cells was randomly generated. Furthermore, the expression profile of this cell type was then mixed with the background mixture in varying proportions ranging from 0% to 100% (Methods). Testing results indicated that the spiked proportions could be precisely measured even when the target compound was present at low concentrations (Fig. 2b), suggesting its good performance for distinguishing rare cell subtypes in the mixtures. Moreover, given the application of ImmuCC for tumor tissues, a simulated dataset was designed to mimic the tumor immune microenvironment. The relative fractions of major immune cell types remained similar to those of the real immune compositions even when the proportion of tumor contents reached 95% (Fig. 2c), which highlighted the tolerance of ImmuCC to the noise of unknown content and also indicated its potential for predicting tumor infiltrated leukocytes.

### Performance on enriched immune cells

**Figure 3:**
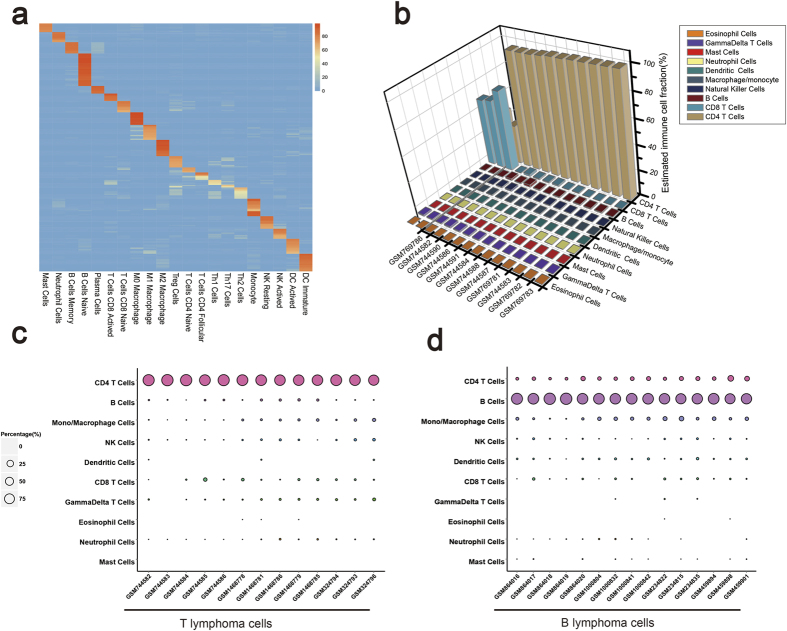
Application of the ImmuCC model on tissue samples. (**a**) Heatmap of deconvolution on 223 arrays of purified immune cells. (**b**) Performance on single positive (SP) thymocyte T cell samples. (**c**) Evaluation of ImmuCC in T-cell acute lymphoblastic leukemia (T-ALL). (**d**) Evaluation of ImmuCC in B-cell leukemia cells. The x-axis labels (**c,d**) was the serious number of sample, the color of the dot represents different cell types, the area of the dot was the proportion of each cell type.

One possible use for ImmuCC is the enrichment of pure samples of immune cells. To test the model performance in this situation, the expression profiles of both purified leukocytes and thymic single positive (SP) T cells were tested. A total of 223 samples of enriched immune cell types profiled on Mouse Genome 430 2.0 platform were used. The predicted proportions of most of the samples were consistent with the expected dominant compositions (Fig. 3a) although some confusion was also observed between some subtypes, like CD8 T cells and CD4 T cells. The confused subtypes could still be classified into the same major cell types (Fig. 3a). Samples profiled on other platforms, including Illumina and Agilent, were also evaluated using this model. Similarly, 18 samples with sorted normal thymic single positive T cells including SP CD4 T cells along with SP CD8 T cells were used for further model assessing. As demonstrated in Fig. 3b, almost all CD4 + SP T cell samples were reasonably predicted with over 97.07% ± 1.52% CD4 T cells. Nonetheless, three SP CD8 T cell samples still made up approximately 40% of all CD4 T cells present.

### Performance on leukemia cell samples

For better evaluation of the performance of ImmuCC, the model was used on leukemia cell samples, including T-cell acute lymphoblastic (T-ALL) and B leukemia. For 13 T-ALL samples, CD4 T cells were predicted to be the dominant cell type with average proportion as 89.92% ± 5.23% (Fig. 3c). Next, among B leukemia cell samples, the calculated fraction of B cells was 82.18% ± 5.85% (Fig. 3d). The predicted dominant CD4 T cells in T-ALL and dominant B cells in B leukemia were consistent with existing knowledge regarding these two typical blood diseases. The good consistency of the prediction and observation among leukemia cells highlighted the suitability of the current model, even for abnormal leukocytes.

### Comparison of flow cytometry analysis in four immune tissues

**Figure 4:**
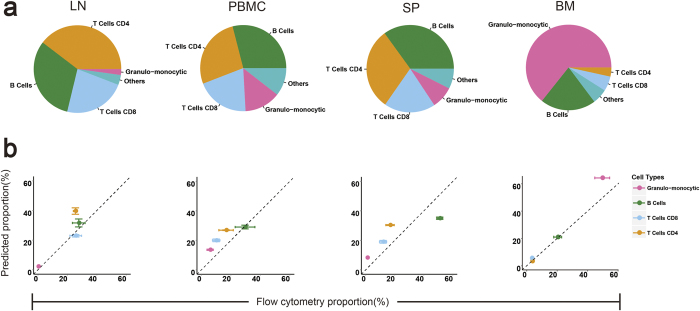
Comparison of ImmuCC and cytometry analysis in four different normal mouse tissues. Four immune tissues consist of bone marrow (BM), spleen (SP), peripheral blood mononuclear cell (PBMC) and lymph node (LN). (**a**) Five major summarized cell types from ImmuCC were shown as pie chart. (**b**) Correlations of ImmuCC and flow cytometry for Granulo-monocytic cells, T cells CD4, T cells CD8 and B cells in four immune organs.

To determine whether ImmuCC can be used to calculate the immune cell compositions of normal tissues, expression profiles were established for three immune organs and one cell type, collected in-house, mouse spleens (SP), lymph nodes (LN), and bone marrow (BM), which are known to be major sources of immune cells, and peripheral blood mononuclear cells (PBMC). The proportions of the dominant types of immune cells in these four categories were closely consistent with the well-accepted range of data (Fig. 4a and Supplementary Fig. S4). For example, the granulo-monocytic cells made up of the largest proportion of immune cell types in BM, accounting for 64.28% ± 1.56% of the total, 10.20% ± 1.36% of monocytes, and 20.97% ± 0.57% of B cells. Similarly, consistent immune cell compositions were observed in both PBMC and SP samples, including approximately 31.99% ± 3.41% B cells, 19.50% ± 0.97% CD8 T cells, 28.60% ± 1.90% CD4 T cells, and 5.54% ± 1.01% NK cells. As with LN samples, there were three main cell types, including B cells (31.59% ± 2.68%), CD4 T cells (39.63% ± 2.16%), and CD8 T cells (22.94% ± 0.83%). In summary, the dominant immune cell types in those four immune organs are primarily in the resting state, including both naive and memory immune cells. They made up almost 70% of the resting immune cells in the SP, PBMC, and LN samples. Similar results were observed when ImmuCC was used on public datasets of these four types of mouse immune tissues (Supplementary Fig. S5).

To further validate the performance of ImmuCC, flow cytometry technology was used to measure the proportions of four major cell types in mouse immune tissues, including Granulo-monocytic cells, CD4 T cells, CD8 T cells, and B cells (Fig. 4b and Supplementary Fig. S6). In the BM, SP, LN, and PBMC samples, good consistency was observed between the predicted fraction and the measured one for all four major immune cell types. Altogether, the high correlation between the results of ImmuCC and flow cytometry analysis not only confirmed the accuracy of the model, but also indicated the applicability of our model for normal mouse tissues.

### Application of ImmuCC in mouse tumor tissues

**Figure 5:**
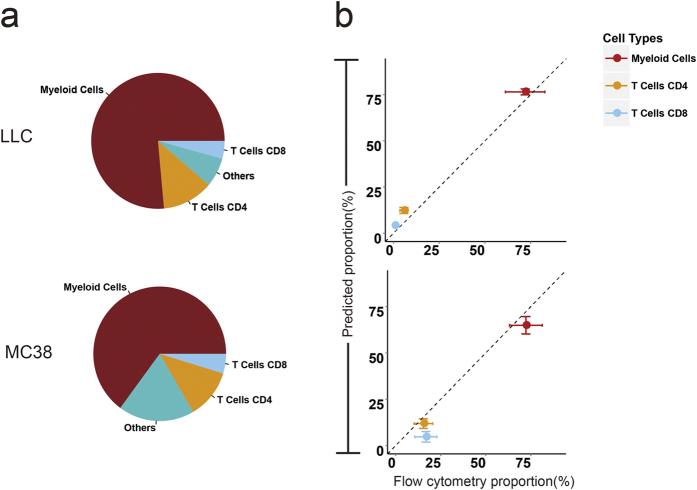
Comparison of ImmuCC and cytometry analysis in two tumor tissues. Two tumor tissues consist of lewis lung cancer (LLC) and murine colon carcinoma cell line (MC38). (**a**) Four major summarized cell types from ImmuCC were shown as pie chart. (**b**) Correlations of ImmuCC and flow cytometry for myeloid cells, T cells CD4 and T cells CD8 in two tumor tissues.

Next, ImmuCC was used to deconvolve the immune compositions for three mouse tumor tissues obtained from subcutaneously implanted histologically distinct mouse cell lines, including Lewis lung cancer cell line (LLC), murine colon carcinoma cell line (MC38), and the T cell lymphoma cell line (EL4). In LLC samples, the dominant cell type was myeloid derived cells (CD45+ CD11b+) including mono/macrophages, dendritic cells, and granulocytes (76.47% ± 1.58%), which also made up the largest proportion (64.88% ± 4.69%) of immune cells in MC38 samples (Fig. 5a and Supplementary Fig. S7). Besides, nearly 30–50% CD4 T cells were Treg cells that were consistent with existing knowledge. However, in EL4 samples, CD4 T cells possessed the dominant part (62.71% ± 2.65%) (Supplementary Fig. S7). Strong consistency was observed among three LLC samples and three MC38 samples, while a slight variation was observed among three EL4 samples, which largely accounted for CD4 memory T cells and follicular T cells (Supplementary Fig. S7). As with normal immune tissues, flow cytometry was also used to measure the proportion of myeloid derived cells, CD4 T cells, and CD8 T cells in LLC and MC38. The measured proportions were good consistent with the results predicted using the ImmuCC model (Fig. 5b). This consistency highlighted the potential usefulness of the ImmuCC model in complex tumor tissues.

## Discussion

In this study we developed an immune composition prediction model named ImmuCC which is tailored to mouse transcriptome data generated from Mouse Genome 430 2.0 platform. Although several methods have been described to dissect the human tissue compositions based on omic data, ImmuCC is still the first such model for mouse tissues thus far. The model has been tested in many types of simulated mixtures, enriched immune cells and different tumor tissues. The predicted results showed good robustness and accuracy in simulated data and in-house generated tissue datasets. The broad scope of potential applications of ImmuCC model was consistent with the important role of mouse models in the study of the immune mechanisms associated with disease. Knowledge of tissue-specific composition of immune cell populations would provide a systematic context and profile for the diagnosis and prognostic of diseases, such as tumors.

Although good performance has been shown, the ImmuCC model still has room for improvement. CD4 T cells and CD8 T cells are the two main T cell subtypes that affect humoral and cellular immune responses. In theory, ImmuCC model should be able to distinguish these two kinds of T cells precisely. However, the testing results indicated mutual interference between CD8 and CD4 T cells (Fig. 3a and b). One possible explanation is that there are large numbers of heavily expressed signature genes shared by both CD8 and CD4 T cells (Fig. 1c). In this way, excessive numbers of shared genes might hamper efforts to correctly distinguish these two subtypes because the mixture was deconvolved with the whole input signature matrix. One potential method of optimization would be to integrate the present algorithm with a hierarchical refinement approach to enhance the performance in different courses of cell subtypes. Another potential problem comes from the microarray data, in which some subtype-specific genes were alternatively spliced into different isoforms that were undetectable in the current microarray platform. This phenomenon may offer another direction for improvements to existing methods through the use of RNA-Seq transcriptome data to predict the composition of the immune cell population.

Tumor-infiltrating immune cells have long been recognized as a key factor for disease prognosis^19^. In this study, relatively proportions of various types of immune cells were determined for both T-ALL and B leukemia using ImmuCC. In all leukemia cell samples, good concordance was observed among samples collected from different labs. ImmuCC was used on subcutaneously implanted tumors, and three major types of immune cells were detected, monocyte/macrophages, cytotoxic CD8+ lymphocyte cells, and CD4+ T cells, which is consistent with previous findings. For example, T regulatory cells tend to accumulate in the tumor microenvironment and play an important role in suppressing the host anti-tumor immune response^20^. NK cells, however, are potent cytotoxic lymphocytes that can accumulate and mediate the immune response against tumor cells in both innate and adaptive immune response^21^. The current model can be further improved to better resolve different types and subtypes of cytotoxic immune cells, including CD8+ T cells and gamma delta T cells.

The performance of ImmuCC relies primarily on the input signature matrix. In current setting, public datasets for immune cells were collected from different sources and tissues. However, immune cells in different tissues may present differential expression profiles. For example, macrophages can take various forms, including Kuepfer cells, alveolar macrophages, microglia, and others, in different tissues^22^. Tissue-microenvironment-induced variations in gene expression have also been observed even for immune-cell-specific genes^23^, which highlight the importance of the development of tissue-specific prediction model for immune cell compositions. Until now, the ImmuCC model has been built from existing public datasets profiled on Affymetrix platform. The good performance of ImmuCC in samples profiled on both Agilent and Illumina array platforms was validated (Supplementary Table S1). This further indicates its suitability for widespread use in different array platforms. With the widespread application of high-throughput next-generation sequencing, RNA-seq transcriptome-based models are also in urgently demand. However, for the moment, the RNA-seq based transcriptome data of single immune cells in public databases are not sufficient to build reasonable models for the prediction of immune cell compositions. In this way, the generation of sets of high-quality RNA-seq-based transcriptome datasets covering all known major types of immune cells would be very important for the model building process.

## Methods

### Brief summary of ImmuCC method

Briefly, ImmuCC is a deconvolution model to find the relative proportion of immune cell types based on the hypothesized linear relationship between mixed expression profile in tissue samples and the expression profile in isolated cell types. The object of deconvolution is to find the solution of a convolution equation in the form: Ax = B. Thus, with a nu–support vector regression (ν-SVR), our objective is to discover a hyperplane that fits as many data points as possible within a specified distance. Those genes selected as support vectors from the signature matrix were kept for later calculation. Three main steps were included in our ImmuCC model. The first step is to perform quantile normalization on the pre-processed data, such as the input sample expression data. The second step is parameter selection. Here a ν-SVR model with a linear kernel were tested with different values of ν and the one with the lowest root mean square error (RMSE) was kept for later calculation. Finally, the immune proportions in each sample were calculated based on these optimized parameters.

### Schematics of methodology development

Six major steps were included in the methodological framework of ImmuCC (Supplementary Fig. S8). First, raw microarray data were collected from public databases and preprocessed to keep 25 immune cell types in the model (Supplementary Fig. S2). Second, significant differentially expressed genes for each immune cell type was selected to generate the signature matrix including 511 genes. Third, the proportions of 25 cell types were deconvolved with the linear SVR approach. Fourth, multiple simulated expression datasets were constructed and tested for the ImmuCC model. Fifth, the ImmuCC model was used for testing the expression profile of enriched immune cell or organs. Sixth, the ImmuCC model was assessed with the flow cytometry analysis in mouse tissues and tumor tissues.

### Microarray data resource and preprocessing

Raw expression profiles of normal mouse immune cells, non-hematopoietic tissues and tumor tissues performed under Affymetrix Mouse Genome 430 2.0 platform were collected and downloaded from the Gene Expression Omnibus (GEO)^24^ and ArrayExpress^25^. The CEL files were normalized with “frma” package in bioconductor^26^. Those arrays with a median GNUSE values <1.25 were considered to be of good quality and retained for latter analysis. Probesets were then converted into HUGO gene symbols using a custom chip definition file (Brainarray version 18). The raw signal intensities of Agilent arrays were download from GEO and analyzed with limma using “normexp” background correction and quantile normalization. Samples profiled on Illumina platforms were downloaded directly as normalized expression matrix from GEO DataSets.

### Construction of signature matrix

To select datasets necessary for evaluation of differential gene expression (DEGs), hierarchical clustering analysis with Pearson correlation coefficient was performed and Principal Component Analysis (PCA) was conducted to evaluate the similarities of different immune cell samples. For each cell type, clusters with the largest proportion of samples were retained (Supplementary Fig. S1). Furthermore, the levels of expression of cell-type-specific marker genes in those clusters were manually confirmed. Finally, 114 samples from which 25 immune cell types that can be significantly distinguished were selected and kept for the next calculation of DEGs (Supplementary Fig. S9). The bioconductor “limma” package was used to compare differential genes of each cell type against all other cell types^27^ Genes with an adjusted *P* value < 0.05 were considered significantly different.

To prevent interference from tissue-specific cells, genes that were highly expressed in the non-hematopoietic tissues were filtered out using the enrichment score (ES), which will be introduced in a subsequent paper. For genes with ES >0, the fractions of non-hematopoietic tissues or cell types were calculated further. Genes with non-hematopoietic fraction >0.05 were regarded as non-hematopoietic genes^28^. Then, significantly expressed hematopoietic genes were ordered by decreasing fold changes for each cell type. The top n significantly expressed genes for each cell type were selected and merged into a matrix covering in total 25 immune cell types. To determine the optimal n, the system was run for 1 to 44 iterations (the largest value in our dataset) to identify the signature matrix with the minimal conditional number^14^. The linear function with a lower conditional number tended to be less sensitive to the change of the input vector, which indicated that signature matrixes with a lower conditional numbers would be more tolerant to the variation of expression profile. Finally, the conditional number was found to be lowest, 21.95, when n was 44. In total, 511 genes were included for 25 immune cell types to form the available signature matrix.

### Enrichment score for filtering non-hematopoietic genes

Raw microarray CEL files including 120 different mouse tissues and cell lines profiled in the Affymetrix Genome 430 2.0 platform were mined and downloaded from GEO. Each tissue or each cell type was compared to the other 119 samples individually. Thus, a total of 119 linear model coefficients for each gene in each group were generated and collected. Because the linear model coefficient is associated with the difference between two groups, the sum of all linear model coefficients with a q value <0.05 served as the enrichment score for each gene in each type of tissue.

### Evaluation of the model in simulated datasets

To evaluate the performance and robustness of the ImmuCC model, two additional simulated datasets were generated, one with a mixture of several types of immune cells and another with a mixture of immune cells and the contents of tumors.

For each of 25 immune cells listed in the signature matrix, a background mixture consisting of the other 24 immune cells was randomly created. Then the expression profile of the target cell type was added into the background mixture in equal-sized increments ranging from 0% to 100%. Fractions of the spiked cell types were then calculated and compared to the concentrations in the real mixtures.

To generate the mixture of immune cell types in a tumor context, an expression profile for an immune mixture of known, random composition was first produced, randomly. Then, this immune mixture was combined with the expression profile of lung tumor cell line sample with different concentrations. Lung tumor contents were added to the immune mixture in even increments ranging from 0% to 90%, and further in 1% increments of ranging from 91% to 99%. The predicted results were compared and evaluated using the known immune compositions.

### Experimental design and testing

Mouse T cell lymphoma, colon carcinoma, and lung carcinoma cells, EL4, MC-38, and LLC-JSP-t2 were cultured in RPMI-1640 medium supplemented with 10% (v/v) fetal bovine serum (FBS; Invitrogen, Carlsbad, CA, U.S.) at 37 °C in a humidified atmosphere containing 5% CO_2_. Six-to-eight-week-old C57BL/6 mice were purchased from Shanghai SLAC Laboratory Animal Co. All mice were maintained in a barrier facility at Animal Center of Soochow University. To evaluate the immune cell compositions of normal mouse immune organs, 12 mice from four immune organs, including the spleen (SP), bone marrow (BM), lymph nodes (LN), and Peripheral blood mononuclear cell (PBMC), were isolated using routine experimental methods. To evaluate the tumor infiltrating lymphocytes, 9 mice were randomly divided into 3 groups and implanted subcutaneously (s.c.) with EL4 (2.0*10^5^/mice), MC-38 (1.0*10^6^/mice), and LLC-JSP-t2 (2.0*10^5^/mice) cells, respectively. All mice were sacrificed and tumor tissues were collected when the tumor volume reached 100 mm^3^. The tissues were ground up with liquid nitrogen and the lysates were stocked using Trizol for subsequent array analysis. Tissues from each group were subjected to RNA isolation and hybridization with Affymetrix Mouse Genome 430 2.0 Array, performed by Capitalbio Technology Corporation. Microarray data pre-processing was performed as described above. All raw data were deposited in GEO (*ID: to be determined*).

### Flow cytometry analysis

Flow cytometry analyses were performed to evaluate the quantity of immune cells in different tissues. Briefly, fresh isolated immune organs and tumor tissues were dissociated into single cells and stained with the correspondent antibodies. Four immune organs were stained with surface antibodies to label CD4 T cells (CD3+ CD4+), CD8 T cells (CD3+ CD8+), B cells (CD3-CD19+), and Granulo-monocytic cells (CD11b+ Gr-1+) and tumor tissues were stained with surface antibodies to label Myeloid cells (CD45+ CD11b+), CD4 T cells (CD45+ CD3+ CD4+) and CD8 T cells (CD45+ CD3+ CD8+). The stained samples were then acquired with Attune Nxt flow cytometry (Life Technology). Finally, the flow cytometry data were analyzed with FlowJo software.

### Statement

All procedures were carried out in accordance with the relevant guidelines, and all experimental protocols were approved by the Chinese Academy of Medical Sciences.

### Availability of ImmuCC

Codes and scripts of this model were written in R version 3.02 and bioconductor version 2.13^29^ and are available in (GitHub: https://github.com/chenziyi/ImmuCC).

## Additional Information

**How to cite this article**: Chen, Z. *et al*. Inference of immune cell composition on the expression profiles of mouse tissue. *Sci. Rep.* **7**, 40508; doi: 10.1038/srep40508 (2017).

**Publisher's note:** Springer Nature remains neutral with regard to jurisdictional claims in published maps and institutional affiliations.

## Supplementary Material

Supplementary Dataset 1

Supplementary 1

## References

[b1] Man Y. G. . Tumor-infiltrating immune cells promoting tumor invasion and metastasis: existing theories. Journal of Cancer 4, 84 95, doi: 10.7150/jca.5482 (2013). 23386907 PMC3564249

[b2] Monticelli L. A. . Innate lymphoid cells promote lung-tissue homeostasis after infection with influenza virus. Nature immunology 12, 1045–1054, doi: 10.1031/ni.2131 (2011). 21946417 PMC3320042

[b3] Mattson D. L. Infiltrating immune cells in the kidney in salt-sensitive hypertension and renal injury. American journal of physiology. Renal physiology 307, F499–508, doi: 10.1152/ajprenal.00258.2014 (2014). 25007871 PMC4154114

[b4] de Lange-Brokaar B. J. . Synovial inflammation, immune cells and their cytokines in osteoarthritis: a review. Osteoarthritis and cartilage/OARS, Osteoarthritis Research Society 20, 1484–1499, doi: 10.1016/j.joca.2012.08.027 (2012). 22960092

[b5] Perlman R. L. Mouse models of human disease: An evolutionary perspective. Evolution, medicine, and public health 2016, 170–176, doi: 10.1093/emph/eow014 (2016). PMC4875775 27121451

[b6] Liu J., Blake S. J., Smyth M. J. & Teng M. W. Improved mouse models to assess tumour immunity and irAEs after combination cancer immunotherapies. Clinical & translational immunology 3, e22, doi: 10.1038/cti.2014.18 (2014). 25505970 PMC4232074

[b7] Yu Y. R. . A Protocol for the Comprehensive Flow Cytometric Analysis of Immune Cells in Normal and Inflamed Murine Non-Lymphoid Tissues. PloS one 11, e0150606, doi: 10.1371/journal.pone.0150606 (2016). 26938654 PMC4777539

[b8] Watkins S. K., Zhu Z., Watkins K. E. & Hurwitz A. A. Isolation of immune cells from primary tumors. Journal of visualized experiments : JoVE, e3952, doi: 10.3791/3952 (2012). PMC3471300 22733225

[b9] Basa R. C. . Decreased Anti-Tumor Cytotoxic Immunity among Microsatellite-Stable Colon Cancers from African Americans. PloS one 11, e0156660, doi: 10.1371/journal.pone.0156660 (2016). 27310868 PMC4911070

[b10] Bergmann B. . Memory B cells in mouse models. Scandinavian journal of immunology 78, 149–156, doi: 10.1111/sji.12073 (2013). 23679222

[b11] Jablonski K. A. . Novel Markers to Delineate Murine M1 and M2 Macrophages. PloS one 10, e0145342, doi: 10.1371/journal.pone.0145342 (2015). 26699615 PMC4689374

[b12] Shen-Orr S. S. & Gaujoux R. Computational deconvolution: extracting cell type-specific information from heterogeneous samples. Current opinion in immunology 25, 571–578, doi: 10.1016/j.coi.2013.09.015 (2013). 24148234 PMC3874291

[b13] Gaujoux R. & Seoighe C. CellMix: a comprehensive toolbox for gene expression deconvolution. Bioinformatics 29, 2211–2212, doi: 10.1093/bioinformatics/btt351 (2013). 23825367

[b14] Abbas A. R., Wolslegel K., Seshasayee D., Modrusan Z. & Clark H. F. Deconvolution of blood microarray data identifies cellular activation patterns in systemic lupus erythematosus. PloS one 4, e6098, doi: 10.1371/journal.pone.0006098 (2009). 19568420 PMC2699551

[b15] Gong T. . Optimal deconvolution of transcriptional profiling data using quadratic programming with application to complex clinical blood samples. PloS one 6, e27156, doi: 10.1371/journal.pone.0027156 (2011). 22110609 PMC3217948

[b16] Qiao W. . PERT: a method for expression deconvolution of human blood samples from varied microenvironmental and developmental conditions. PLoS computational biology 8, e1002838, doi: 10.1371/journal.pcbi.1002838 (2012). 23284283 PMC3527275

[b17] Zhong Y., Wan Y. W., Pang K., Chow L. M. & Liu Z. Digital sorting of complex tissues for cell type-specific gene expression profiles. BMC bioinformatics 14, 89, doi: 10.1186/1471-2105-14-89 (2013). 23497278 PMC3626856

[b18] Newman A. M. . Robust enumeration of cell subsets from tissue expression profiles. Nature methods 12, 453–457, doi: 10.1038/nmeth.3337 (2015). 25822800 PMC4739640

[b19] Pages F. . Immune infiltration in human tumors: a prognostic factor that should not be ignored. Oncogene 29, 1093–1102, doi: 10.1038/onc.2009.416 (2010). 19946335

[b20] Joshi N. S. . Regulatory T Cells in Tumor-Associated Tertiary Lymphoid Structures Suppress Anti-tumor T Cell Responses. Immunity 43, 579–590, doi: 10.1016/j.immuni.2015.08.006 (2015). 26341400 PMC4826619

[b21] Waldhauer I. & Steinle, NK cells and cancer immunosurveillance. Oncogene 27, 5932–5943, doi: 10.1038/onc.2008.267 (2008). 18836474

[b22] Davies L. C. & Taylor P. R. Tissue-resident macrophages: then and now. Immunology 144, 541–548, doi: 10.1111/imm.12451 (2015). 25684236 PMC4368161

[b23] Lavin Y. . Tissue-resident macrophage enhancer landscapes are shaped by the local microenvironment. Cell 159, 1312–1326, doi: 10.1016/j.cell.2014.11.018 (2014). 25480296 PMC4437213

[b24] Clough E. & Barrett T. The Gene Expression Omnibus Database. Methods in molecular biology 1418, 93–110, doi: 10.1007/978-1-4939-3578-9_5 (2016). 27008011 PMC4944384

[b25] Parkinson H. . ArrayExpress–a public database of microarray experiments and gene expression profiles. Nucleic acids research 35, D747–750, doi: 10.1093/nar/gkl995 (2007). 17132828 PMC1716725

[b26] McCall M. N. & Irizarry R. A. Thawing Frozen Robust Multi-array Analysis (fRMA). BMC bioinformatics 12, 369, doi: 10.1186/1471-2105-12-369 (2011). 21923903 PMC3180392

[b27] Ritchie M. E. . limma powers differential expression analyses for RNA-sequencing and microarray studies. Nucleic acids research 43, e47, doi: 10.1093/nar/gkv007 (2015). 25605792 PMC4402510

[b28] Benita Y. . Gene enrichment profiles reveal T-cell development, differentiation, and lineage-specific transcription factors including ZBTB25 as a novel NF-AT repressor. Blood 115, 5376–5384, doi: 10.1182/blood-2010-01-263855 (2010). 20410506 PMC2902135

[b29] Gentleman R. C. . Bioconductor: open software development for computational biology and bioinformatics. Genome biology 5, R80, doi: 10.1186/gb-2004-5-10-r80 (2004). 15461798 PMC545600

