# Corrigendum: Inference of immune cell composition on the expression profiles of mouse tissue

**DOI:** 10.1038/srep45416

**Published:** 2017-03-24

**Authors:** Ziyi Chen, Anfei Huang, Jiya Sun, Taijiao Jiang, F. Xiao-Feng Qin, Aiping Wu

Scientific Reports 7: Article number: 4050810.1038/srep40508; published online: 01 13 2017; updated: 03 24 2017

The authors regret that previous work cited in reference 18 reporting the CIBERSORT method was not properly acknowledged for their contribution to the development of methodological framework utilized in this work.

In the Introduction section,

“Here, a mouse-specific algorithm called ImmuCC was developed to infer the proportions of 25 types of immune cells with 511 selected signature genes from mouse tissue microarray datasets”.

should read:

“Here, ImmuCC mainly includes a mouse-specific leukocyte signature matrix that was developed to infer the proportions of 25 mouse immune cell types using the CIBERSORT deconvolution framework^18^. ImmuCC consists of 511 immune signature genes that were selected from mouse tissue microarray datasets to optimize deconvolution performance based on a previously described approach^18^”.

In the Results section, under subheading ‘**An overview of the ImmuCC model**’,

“Here a computational model named ImmuCC was developed to infer the relatively compositions of 25 immune cell types with 511 signature genes via the linear support vector regression (SVR) approach”.

should read:

“Here a new mouse immune signature matrix was developed to infer the relative composition of 25 mouse immune cell types using CIBERSORT^18^, a gene expression deconvolution approach based on linear support vector regression (SVR)”.

In the Methods section, under subheading ‘**Brief summary of ImmuCC method**’,

“Briefly, ImmuCC is a deconvolution model to find the relative proportion of immune cell types based on the hypothesized linear relationship between mixed expression profile in tissue samples and the expression profile in isolated cell types”.

should read:

“Briefly, ImmuCC leverages a signature matrix optimized for mouse leukocyte deconvolution. Coupled with CIBERSORT^18^, ImmuCC can determine the relative proportion of mouse immune cell types based on the hypothesized linear relationship between mixed expression profile in tissue samples and the expression profile in isolated cell types”.

