# Supplementary material for: Inference of immune cell composition on the expression profiles of mouse tissue
Source: Sci Rep. 2017 Jan 13;7:40508. doi: 10.1038/srep40508 (PMC5233994; doi:10.1038/srep40508)
Supplement: Supplementary 1 [file srep40508-s2.pdf]

Inference of immune cell composition on the expression profiles of mouse tissue

Ziyi Chen[1, †], Anfei Huang[1,†], Jiya Sun[1], Taijiao Jiang[1], F. Xiao-Feng Qin[1,*] and Aiping Wu[1,*]

[1]Center for Systems Medicine, Institute of Basic Medical Sciences, Chinese Academy of Medical Sciences & Peking Union Medical College, Beijing 100005, Suzhou Institute of Systems Medicine, Suzhou, Jiangsu 215123, China;

*To whom correspondence should be addressed. Email: wap@ism.cams.cn (A.W.); fqin1@foxmail.com (F.X.Q.).

†These authors contributed equally to this work.

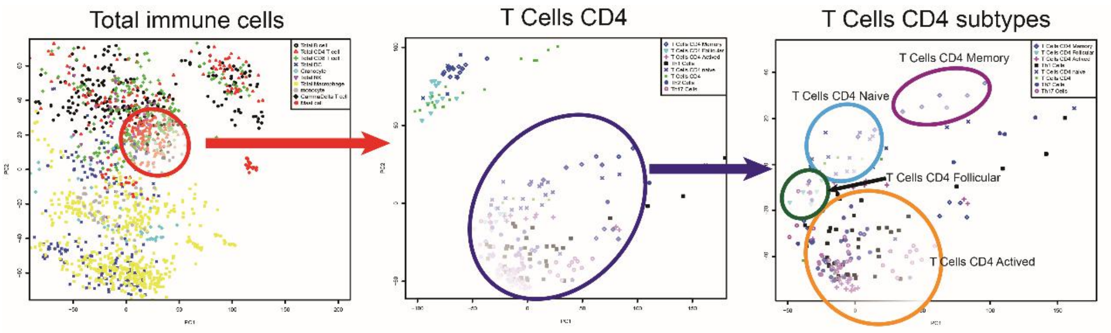

Supplemental figure S1. Dataset selection with PCA. All immune cell datasets were first separated into major cell types, and further into cell subtypes.

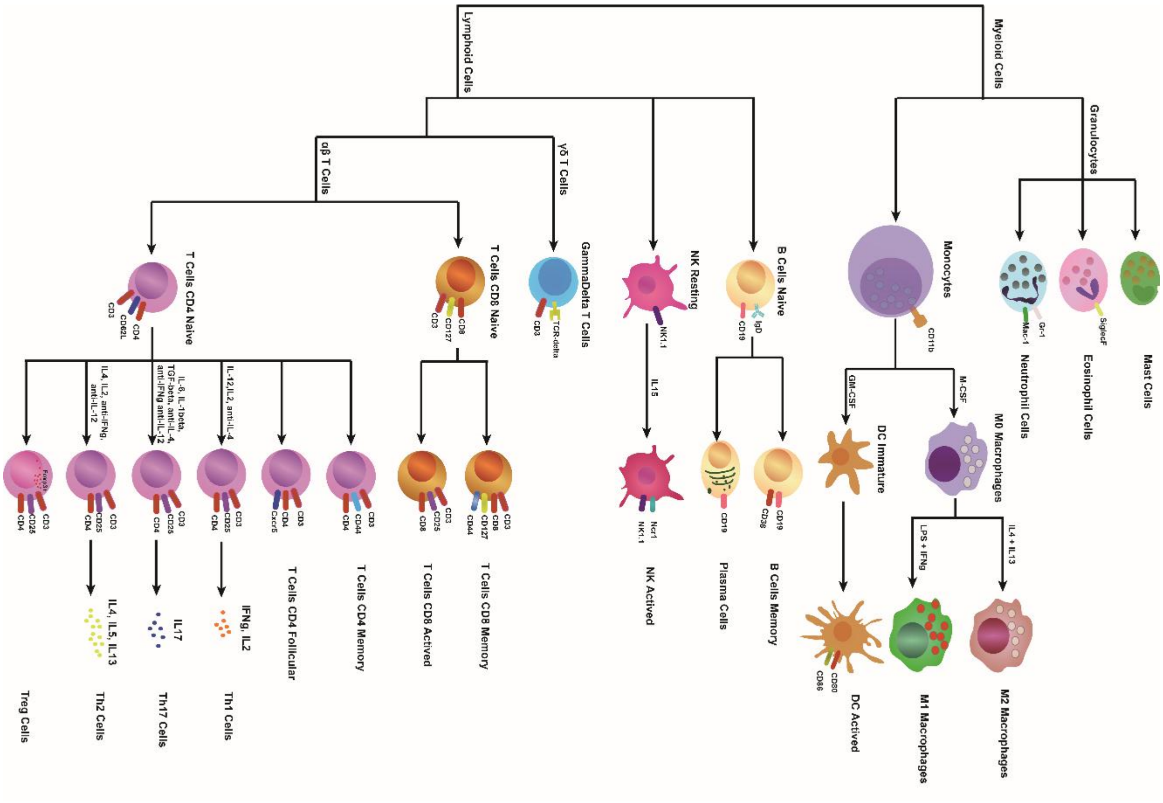

Supplemental figure S2. The distribution of 25 selected immune cell types in the differentiation tree of hematopoietic cells.

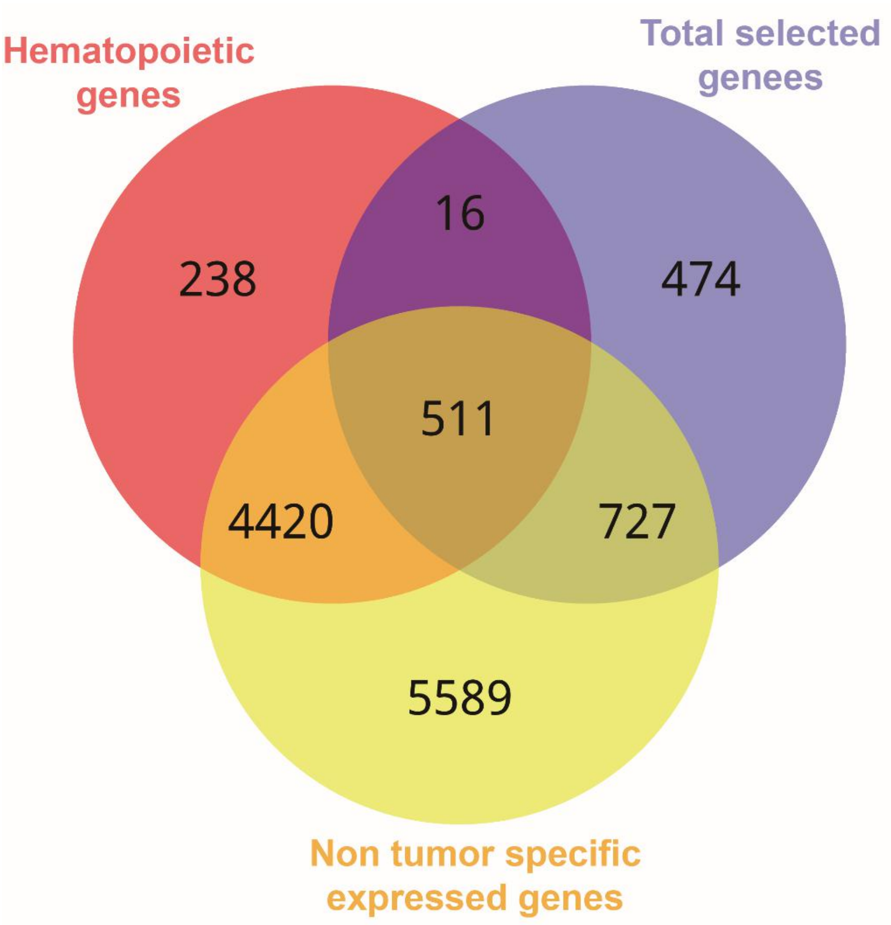

Supplemental figure S3. Venn diagrams for the overlapping genes between total selected differentially expressed genes, hematopoietic genes and non-tumor highly expressed probes.

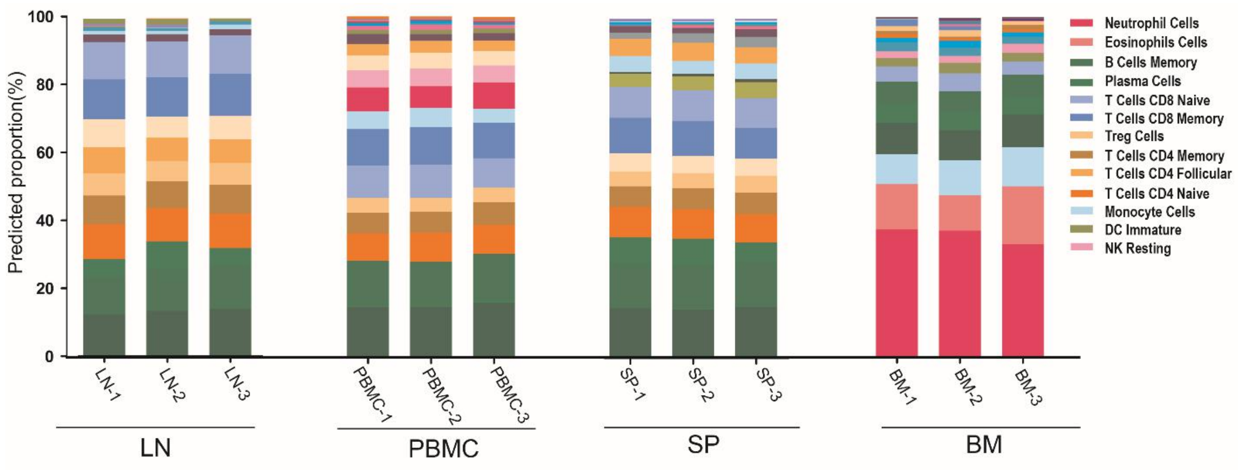

Supplemental figure S4. Estimation of immune cell compositions in four mouse immune tissues collected by ourselves. Four immune tissues consist of bone marrow (BM), spleen (SP), peripheral blood mononuclear cell (PBMC) and lymph node (LN). The cell type compositions were shown as bar chart.

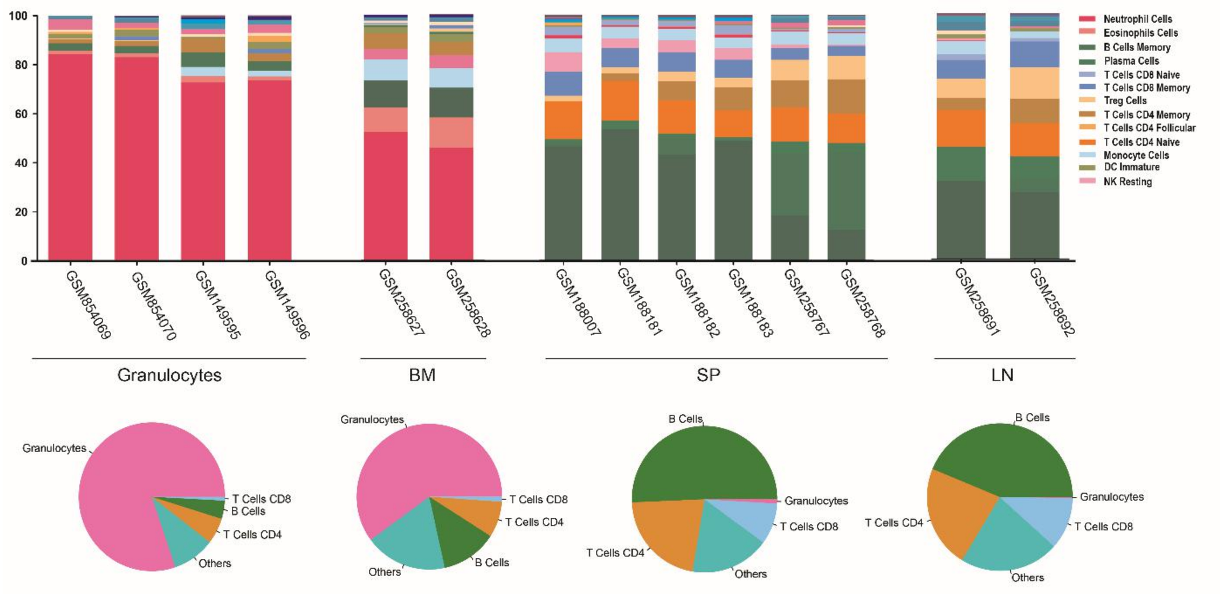

Supplemental figure S5. Estimation of immune cell compositions in four mouse immune tissues from public datasets. Four immune tissues consist of granulocytes bone marrow (BM), spleen (SP) and lymph node (LN). The cell type compositions were shown as bar chart. Five major summarized cell types were shown as pie chart.

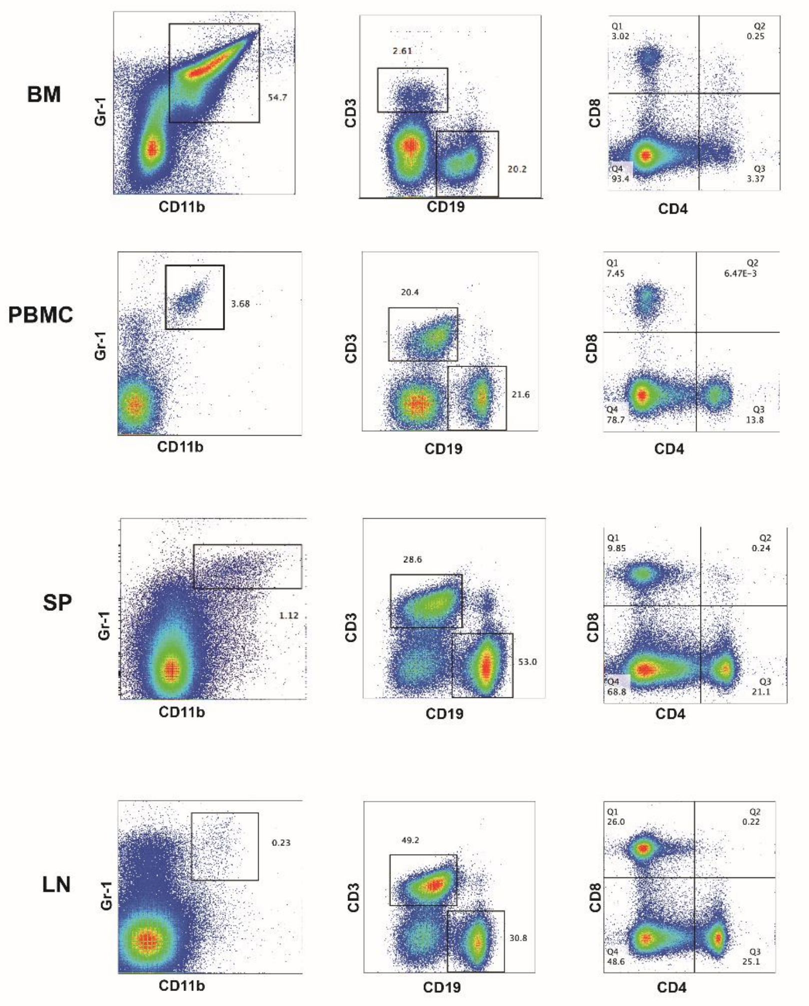

Supplemental figure S6. Gating strategy for flow cytometry scatter plots to analyze the frequency of Granulocytes, T Cells CD4+, T Cells CD8+ and B Cells in four immune organs consist of bone marrow (BM), spleen (SP), peripheral blood mononuclear cell (PBMC) and lymph node (LN).

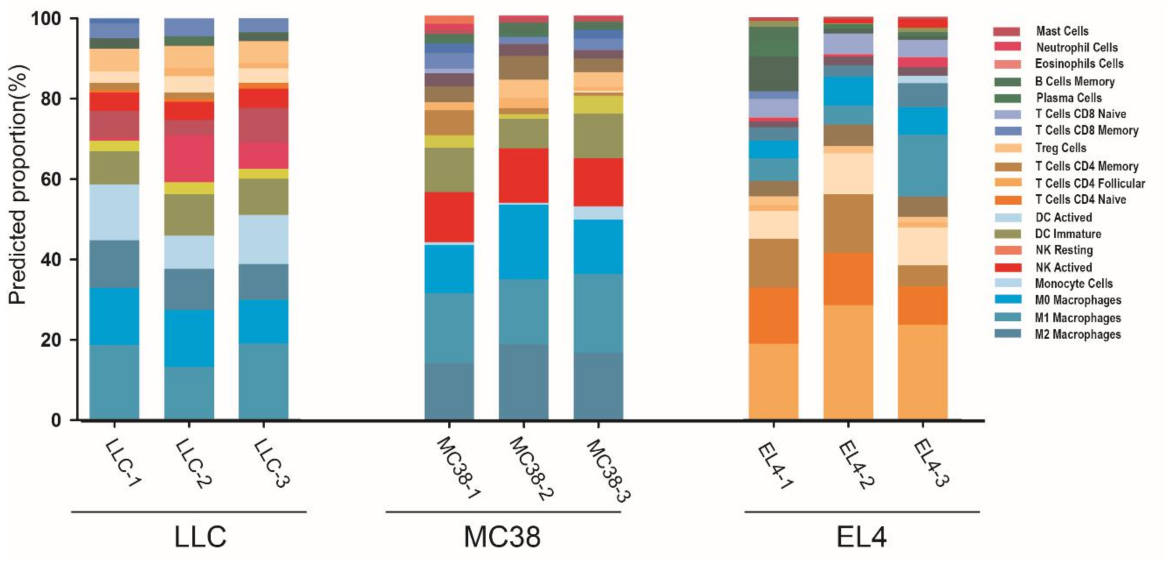

Supplemental figure S7. Estimation of immune cell compositions in three tumor tissues collected by ourselves. Four immune tissues consist of bone marrow (BM), spleen (SP), peripheral blood mononuclear cell (PBMC) and lymph node (LN). The cell type compositions were shown as bar chart.

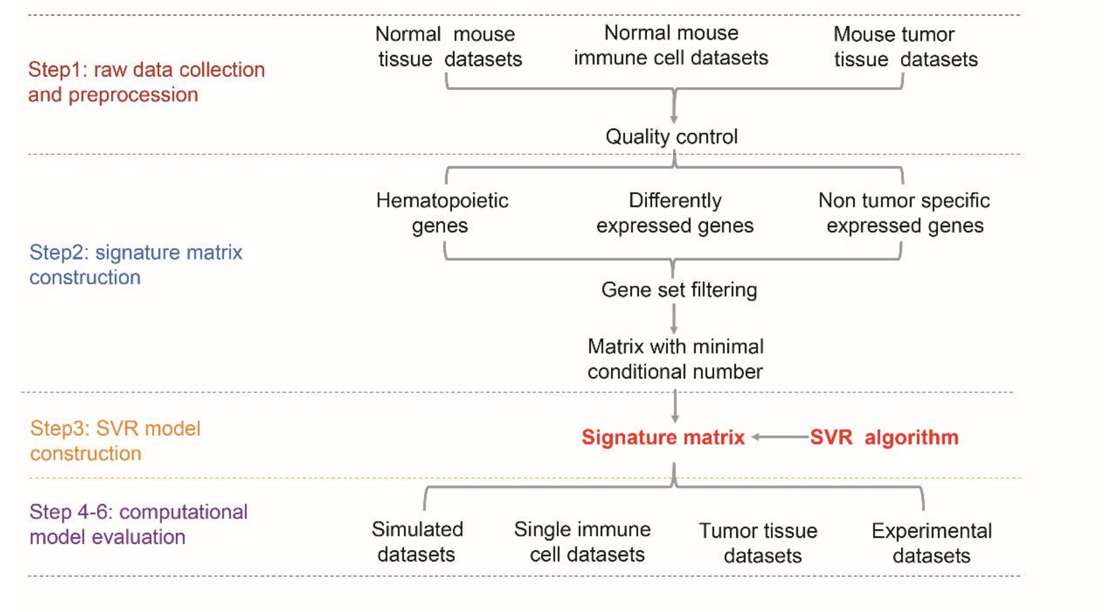

Supplemental figure S8. The schematic of the ImmuCC model. Four main steps consist of data collection, gene set selection, signature matrix construction and model validation.

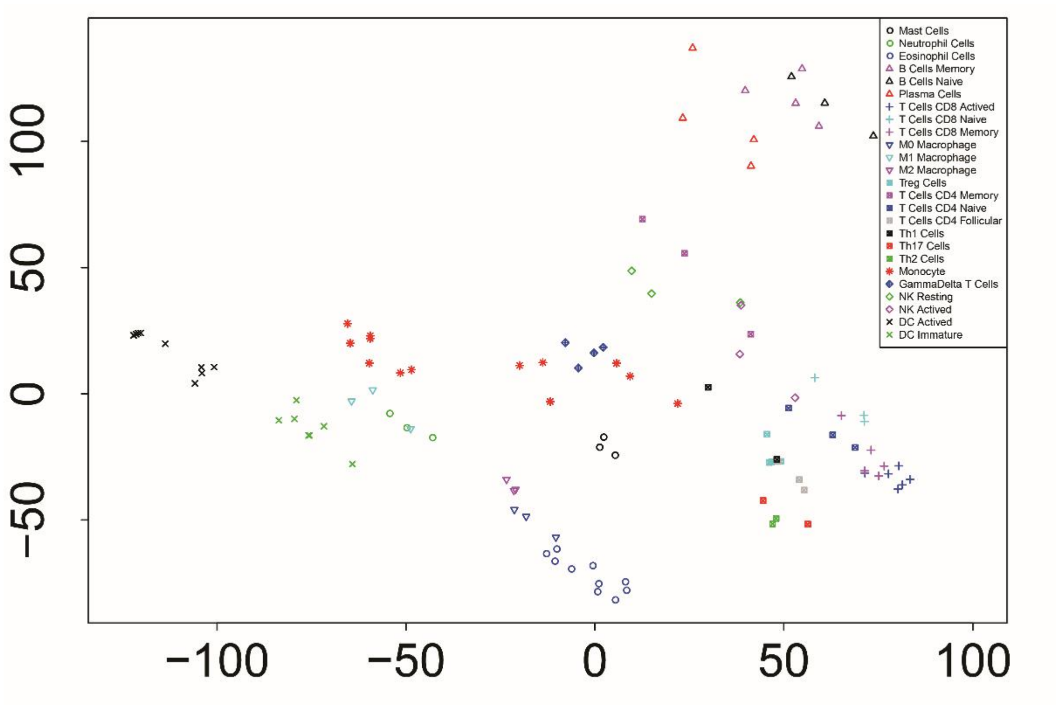

Supplemental figure S9. PCA of 25 selected immune cell types.

Supplementary Table S1 Validation of ImmuCC on Agilent and Illumina platforms. Transcriptomic data profiled on Agilen4x44K, Agilen8x60K and Illumina MouseWG-6 v2.0 were calculated with our model. Immune cell type with the highest fraction in each sample was compared to the known purified cell type and counted as correct if they were matched.

Cell type	Platforms	Data set	Correct number	Total number	Percent correct
Dendritic Cells	Agilen4x44K	GSE43169	2	5	40%
Dendritic Cells	Agilen4x44K	GSE56929	24	24	100%
Macrophages	Agilen4x44K	GSE55693	20	20	100%
Macrophages	Agilen4x44K	GSE71814	6	6	100%
T Cells CD4	Agilen4x44K	GSE57098	12	12	100%

T Cells CD4	Agilen4x44K	GSE58152	9	9	100%
B cells	Agilen8x60K	GSE63391	7	8	88%
B cells	Agilen8x60K	GSE73026	4	4	100%
M1 macrophage	Agilen8x60K	GSE51466	3	3	100%
M2 macrophage	Agilen8x60K	GSE51466	3	3	100%
Macrophages	Agilen8x60K	GSE51466	5	6	83%
Macrophages	Agilen8x60K	GSE58003	2	2	100%
Macrophages	Agilen8x60K	GSE58098	3	3	100%
Macrophages	Agilen8x60K	GSE64473	9	9	100%
Macrophages	Agilen8x60K	GSE73026	8	8	100%
Macrophages	Agilen8x60K	GSE73311	24	24	100%
Macrophages	Agilen8x60K	GSE39660	6	6	100%
Macrophages	Agilen8x60K	GSE80065	6	6	100%
Neutrophils	Agilen8x60K	GSE55995	13	16	81%
T Cells CD4	Agilen8x60K	GSE50149	10	10	100%
T Cells CD4	Agilen8x60K	GSE60356	14	14	100%
T Cells CD4	Agilen8x60K	GSE67465	12	12	100%
T Cells CD4	Agilen8x60K	GSE76693	3	3	100%
T Cells CD8	Agilen8x60K	GSE45673	6	8	75%
B Cells	Illumina MouseWG-6 v2.0	GSE51604	12	12	100%
Dendritic Cells	Illumina MouseWG-6 v2.0	GSE60146	21	21	100%
Macrophages	Illumina MouseWG-6 v2.0	GSE68167	17	18	94%
Neutrophils	Illumina MouseWG-6 v2.0	GSE30887	8	10	80%

NK Cells	Illumina MouseWG-6 v2.0	GSE34237	4	9	44%
T Cells CD4	Illumina MouseWG-6 v2.0	GSE52185	35	35	100%
T Cells CD8	Illumina MouseWG-6 v2.0	GSE44140	12	12	100%
